# Adherence to hydroxyurea, health-related quality of life domains, and patients’ perceptions of sickle cell disease and hydroxyurea: a cross-sectional study in adolescents and young adults

**DOI:** 10.1186/s12955-017-0713-x

**Published:** 2017-07-05

**Authors:** Sherif M. Badawy, Alexis A. Thompson, Jin-Shei Lai, Frank J. Penedo, Karen Rychlik, Robert I. Liem

**Affiliations:** 1 Department of Pediatrics, Division of Hematology, Oncology and Stem Cell Transplant, Ann & Robert H. Lurie Children’s Hospital, Northwestern University Feinberg School of Medicine, 225 E. Chicago Ave., Box #30, Chicago, IL 60611 USA; 2 Department of Medical Social Sciences, Northwestern University Feinberg School of Medicine, 633 N. St Clair, Suite 19-000, Chicago, IL 60611 USA; 3 Stanley Manne Children’s Research Institute, Ann & Robert H. Lurie Children’s Hospital, 225 E Chicago Ave., Chicago, IL 60611 USA; 4 Department of Pediatrics, Division of Hematology and Oncology, Zagazig University Faculty of Medicine, Zagazig, Egypt

**Keywords:** Sickle cell disease, Hydroxyurea, Adherence, Health-related quality of life, Patient reported outcomes, Perceptions, Beliefs

## Abstract

**Background:**

Sickle cell disease (SCD) patients have impaired domains of health-related quality of life (HRQOL). Hydroxyurea is safe and efficacious in SCD; however, adherence is suboptimal, and patients’ perceptions are poorly understood amongst adolescents and young adults (AYA). Study objectives were to: (1) examine patients’ perceptions of SCD and hydroxyurea; and (2) explore the relationship of their perceptions to clinical characteristics, HRQOL domains and hydroxyurea adherence.

**Methods:**

Thirty-four SCD patients on hydroxyurea (≥6 months) participated in a single-institution study. Study measures included Brief-Illness Perceptions Questionnaire, **©**Modified Morisky Adherence Scale 8-items, and Patient Reported Outcomes Measurement Information System (PROMIS®). We assessed the relationship of patients’ perceptions to hydroxyurea adherence using Wilcoxon rank-sum test, the number of hospitalizations using Kruskal-Wallis test, and the number of ED visits, adherence level, HRQOL domain scores using Spearman’s rho correlations. We conducted a sub-analysis in HbSS patients to evaluate the relationship of patients’ perceptions to laboratory markers of hydroxyurea adherence.

**Results:**

Participants were 59% male and 91% Black, and had a median age of 13.5 (range 12–18) years. Participants with ≥4 hospitalizations over 1-year prior (using electronic medical chart review) reported more negative perceptions of SCD-related symptoms and emotional response, and perceived hydroxyurea as less beneficial; all *p*-values ≤0.01. Most participants (74%) reported low hydroxyurea adherence. Participants with higher hydroxyurea adherence perceived more hydroxyurea benefits (*r*_s_ = 0.44, *p* < 0.01) and had better emotional response to SCD (*r*_s_ = −0.44, *p* = 0.01). In a sub-analysis of HbSS patients, perceived benefits of hydroxyurea positively correlated with HbF (*r*_s_ = 0.37, *p* = 0.05) and MCV values (*r*_s_ = 0.35, *p* = 0.05). Participants with more negative perceptions of SCD-related consequences, concerns, and emotional response, and fewer perceived hydroxyurea benefits reported worse fatigue (*r*_s_ = 0.68; *r*_s_ = 0.44; *r*_s_ = 0.74; *r*_s_ = −0.60), pain (*r*_s_ = 0.56; *r*_s_ = 0.54; *r*_s_ = 0.63; *r*_s_ = −0.39), anxiety (*r*_s_ = 0.55; *r*_s_ = 0.58; *r*_s_ = 0.56; *r*_s_ = −0.47), and depression (*r*_s_ = 0.64; *r*_s_ = 0.49; *r*_s_ = 0.70; *r*_s_ = −0.62), respectively, all *p*-values <0.05.

**Conclusions:**

Dynamics influencing hydroxyurea adherence are multifactorial, and understanding patients’ perceptions is critical to overcoming adherence barriers. Patients’ favorable perceptions correlated with greater adherence and better HRQOL domain scores. Prospective evaluation of patients’ perceptions of SCD and hydroxyurea in relation adherence, HRQOL domains and clinical outcomes is warranted.

## Background

Sickle cell disease (SCD) is an inherited red blood cell disorder seen in 100,000 Americans and 1 every 365 African-American births [1]. Patients with SCD encounter a number of complications across their lifespan, such as chronic anaemia, acute and chronic pain, acute chest syndrome, and long-term end-organ damage [2]. Compared to healthy controls, patients with SCD experience significant declines in domains of health-related quality of life (HRQOL) across their lifespan due to the impact of disease-related complications [3]. Over the past 2 decades, treatment options for patients with SCD have included hydroxyurea, chronic transfusions, and stem cell transplantation [4].

There has been accumulating evidence to support the safety, efficacy, and cost effectiveness of using hydroxyurea in paediatric and adult SCD patients with demonstrated benefits related to morbidity, mortality, and domains of HRQOL [5–12]. Nevertheless, hydroxyurea utilization and adherence remain suboptimal in this population [13–18]. Barriers to hydroxyurea adherence include forgetfulness, fear of side effects (e.g. birth defects and cancer risk), limited knowledge about hydroxyurea, inability to obtain refills, and misperceptions of SCD severity [13–23].

Adherence to hydroxyurea is a multi-factorial process that is influenced by parental and patient perceptions about hydroxyurea, SCD severity or both. Thornburg et al. reported that most parents perceived hydroxyurea as a valuable medication that helped their children to have less frequent pain episodes, be more physically active, and miss fewer days of school [14]. However, hydroxyurea acceptance and adherence are influenced by parental and patient perceptions of treatment benefits versus risks, which may depend on how they perceive their disease severity. Earlier reports showed that parents were more willing to accept the risk of hydroxyurea side effects if their children had more severe forms of SCD [20, 24–26]. Nevertheless, patients’ perceptions of SCD and hydroxyurea are not well studied in adolescents and young adults (AYA) with SCD as earlier studies examined predominantly parental perceptions. In addition, the relationship of patients’ perceptions to their HRQOL domain scores and hydroxyurea adherence rates remains unclear. Understanding patients’ and/or parental perceptions of SCD as well as of hydroxyurea safety and efficacy is a key step towards developing behavioral interventions aimed at changing or improving their perceptions in a way that could lead to improvements in adherence rates. Changes in patients’ perceptions of hydroxyurea and its efficacy could also serve as surrogate markers for early changes in hydroxyurea adherence over time.

The objectives of this study were to: (1) examine patients’ perceptions of SCD and hydroxyurea amongst AYA using the Brief Illness Perception Questionnaire (B-IPQ) [27, 28]; and (2) explore the relationship of patients’ perceptions of SCD and hydroxyurea to their demographic and clinical characteristics, domains of HRQOL and adherence to hydroxyurea. We hypothesized that patient perceptions of SCD, hydroxyurea, or both, could be associated with level of hydroxyurea adherence and HRQOL domain scores.

## Methods

### Participants enrollment

We conducted a cross-sectional study using non-probability convenience sampling methodology. We approached AYA who were 12–22 years old, had SCD patients (all genotypes), were English-speaking and on a steady state dose of hydroxyurea, defined as a stable dose for 2 months or more prior to enrollment in the study. Between January 2015 and December 2015, patients were enrolled before or after their scheduled outpatient comprehensive sickle cell clinic or hydroxyurea clinic appointments. Exclusion criteria included patients with SCD on chronic transfusion support, with any haemoglobin disorder other than SCD, or with any cognitive or physical disability.

### Perception measure

We evaluated patients’ perceptions using the brief illness perception questionnaire (B-IPQ), which was adapted to reflect hydroxyurea and SCD [28]. In the adapted B-IPQ, we replaced the word “illness” by “sickle cell disease” and the word “treatment” by “Hydroxyurea”. For example, one of the questions was “How much do you think your hydroxyurea can help your sickle cell disease?” instead of “How much do you think your treatment can help your illness?” In addition, two items were deemed not applicable and/or could be confusing for patients with SCD, and therefore were deleted in the adapted B-IPQ version. The B-IPQ has been extensively utilized in earlier studies and used in patients as young as 8 years of age [27, 28]. The adapted B-IPQ consists of 7 items or dimensions including perceived consequences of SCD, personal control of SCD, control of SCD with hydroxyurea, identity (i.e. SCD-related symptoms), concerns about SCD, understanding of SCD, and emotional response secondary to having SCD. The “Identity” domain will be referred to as “symptoms” throughout the manuscript. Each item uses a scale from 0 to 10 to assess each dimension, and each item is evaluated as a separate subscale. Higher scores indicate stronger perceptions of each dimension of the B-IPQ. In particular, higher B-IPQ scores indicate more negative perceptions of SCD-related consequences, identity (i.e. more perceived symptoms), concerns, and emotional response, but more positive perceptions of personal control, treatment control, and understanding of SCD.

### HRQOL measures

Study participants were asked to complete Patient Reported Outcomes Measurement Information System – Computerized Adaptive Testing (PROMIS®-CAT) measures using an electronic tablet [29]. PROMIS-CAT is a novel application of a comprehensive, item-response theory optimized item bank, which enables precise estimation of a PRO domain while simultaneously minimizing burden to participants [30, 31]. In CAT, items administered are selected based on informant’s previous item responses, using a pre-set computerized algorithm based on individual item information functions. Therefore, the total number of items used in different PROMIS measures vary within and in-between patients. A number of PROMIS® measures have been validated in the paediatric and adult populations [32–35], and have been recently evaluated in SCD [36, 37]. Adolescents (age 12–17 years) were asked to complete paediatric PROMIS®-CAT measures of fatigue, pain interference, physical functioning mobility and upper-extremity, depression, anxiety, and peer relationships. Young adults (age 18–22 years) were asked to complete adult PROMIS®-CAT measures of fatigue, pain interference, physical functioning mobility and upper-extremity, depression, anxiety, and social isolation. PROMIS peer relationships and social isolation measures have different set of questions, however both evaluate patients’ relationships and social support. All paediatric and most adult PROMIS®-CAT measures elicited participants’ responses based on the previous 7 days using 5-point response options ranging from “with no trouble” to “not able to do” for physical functioning measures and from “never” to “almost always” in all other measures. No timeframe was assigned to adult PROMIS physical functioning measure. Higher PROMIS® domain scores indicated better physical functioning (mobility or upper-extremity), and peer relationships, but worse severity for fatigue, pain interference, depression, anxiety, and social isolation. PROMIS®-CAT paediatric and adult measures were scored on a general population based T-score metric with a mean of 50 and a standard deviation of 10 [32, 38].

### Adherence measure

Hydroxyurea adherence was evaluated using the **©**Modified Morisky Adherence Scale 8-items (**©**MMAS-8), which was adapted to reflect hydroxyurea and SCD. In the adapted **©**MMAS-8, we replaced the words “medicine”, “medication” and “pills” with “Hydroxyurea”. **©**MMAS-8 includes 7 yes/no questions and 1 multiple-choice question, and evaluates adherence over the past day and 2 weeks [39, 40]. Total **©**MMAS-8 numerical scores were calculated per the assessment manual. Higher **©**MMAS-8 scores indicated better adherence to hydroxyurea. Based on the total **©**MMAS-8 score, three levels of adherence were also considered: low (0 to <6), medium (6 to <8) and high (8).

### Data collection

Data were directly captured from electronic tablets using two online platforms at Northwestern University Feinberg School of Medicine: a) REDCap for hydroxyurea adherence and perceptions data, supported by the Northwestern University Clinical and Translational Sciences (NUCATS) Institute; and b) the Assessment Center^SM^ (https://www.assessmentcenter.net/) for PROs data, supported by the Department of Medical Social Sciences, Northwestern University. Pilot testing was conducted to ensure tablet and Internet functionality in the outpatient setting. We collected information on SCD-related clinical events (e.g. number of emergency department (ED) visits and/or hospitalization at our institution in the year prior to enrollment), and hydroxyurea indication, dose, and duration of treatment through electronic medical chart review of enrolled participants. One author (SB) conducted chart review using a customized data extraction sheet. We obtained selected laboratory markers at the time of study enrollment, including fetal haemoglobin (HbF) and mean corpuscular volume (MCV).

### Statistical analysis

We used descriptive statistics to report categorical data in frequencies and percentages. All B-IPQ scores were presented as medians, interquartile range, or both. An exploratory analysis was conducted to evaluate the relationship of patients’ perceptions of SCD and hydroxyurea to their demographic and clinical characteristics, domains of HRQOL and adherence to hydroxyurea. We used Wilcoxon rank-sum test to determine if patients’ perceptions (B-IPQ domain scores) were related to different categories of age groups, sex, hydroxyurea adherence level, HbF%, and/or MCV values. We also used Kruskal-Wallis test to assess if patients’ perceptions (B-IPQ domain scores) were related to number of SCD-related hospitalizations. Spearman’s rho (r_s_) correlations were used to examine the relationship of patients’ perceptions (B-IPQ domain scores) to number of ED visits in 1 year prior to study enrollment, hydroxyurea adherence level using **©**MMAS-8 and HRQOL domain scores using PROMIS measures. Participants were categorized according to their age as adolescents [12–17 years] (*n* = 25) and young adults [18–22 years] (*n* = 9); number of hospitalizations in the year prior to study enrollment as none (*n* = 14), 1–3 (*n* = 11), and 4 or more hospitalizations (*n* = 9); and hydroxyurea adherence level as low (*n* = 25) and moderate/high (*n* = 9). Given that most of the evidence in the literature on laboratory response to hydroxyurea is based on studies that included mainly patients with HbSS disease, we conducted a sub-analysis in this population to evaluate the relationship between laboratory markers of hydroxyurea adherence and patients’ perceptions of SCD and hydroxyurea. In HbSS patients only (*N* = 29), patients were categorized according to their median values for HbF% (16%) and MCV (102 fl) to compare HRQOL domain scores across groups. HbF% was low if it was less than 16% (*n* = 15) and high if HbF was equal to or greater than 16% (*n* = 14); and MCV was low if it was less than 102 fl (*n* = 15) and high if MCV was equal to or greater than 102 fl (*n* = 14). PROMIS® T-scores from the sample were analyzed as a group rather than stratified by age. All tests were 2-sided and a *p*-value <0.05 was considered statistically significant. We conducted a complete case analysis where missing variables are dropped from the analysis, leaving only complete cases. Statistical analysis was performed using SAS 9.3 (SAS Institute Inc., Cary, NC).

## Results

### Participants characteristics

A total of 34 patients were approached, and all agreed to participate and were enrolled in the study (100% participation rate). The majority of participants were male (59%), Black (91%), and had a median age of 13.5 years (IQR 12–18) and a mean (SD) age of 14.8 (3) years. Most study participants had homozygous SCD (85%), and had hydroxyurea prescribed for recurrent pain episodes (53%). Almost 60 % of participants had one or more hospitalizations in the year prior to study enrollment with a median length of SCD-related hospital stay of 22 days (IQR 0–87). Table 1 summarizes participants’ demographic, clinical, and laboratory characteristics.

**Table 1:** Patient characteristics

Characteristics	Study cohort (*N* = 34)
Age (years), median (IQR)	13.5 (12–18)
Adolescents (12–17 years), n (%)	25 (73.5)
Young adults (18–22 years), n (%)	9 (26.5)
Male, n (%)	20 (59)
Race/Ethnicity, n (%)	
African American	31 (91)
Hispanics or Latino	3 (9)
Genotype, n (%)	
HbSS	29 (85.3)
HbSC	3 (8.8)
HbSB^0^	2 (5.9)
Indication for hydroxyurea, n (%)	
Recurrent pain	18 (52.9)
Recurrent ACS	2 (5.9)
Recurrent pain and ACS	9 (26.5)
Others^a^	5 (14.7)
Hydroxyurea dose (mg/kg/dose), median (IQR)	33.8 (28.9–35)
Hydroxyurea duration (months), median (IQR)	70.5 (30–100)
Hydroxyurea duration - steady dose (months), median (IQR)	14 (7–22)
Number of SCD-related hospitalizations in 1 year, n (%)	
0	14 (41.2)
1–3	11 (32.3)
4 or more	9 (26.5)
Laboratory markers, median (IQR)	
Fetal haemoglobin (%)	15.1 (9.4–30.7)
Haemoglobin (g/dl)	9.4 (8.5–10)
Red blood cells count (10^6^/ml)	2.6 (2.4–3.1)
Mean corpuscular volume (fl)	101.1 (92.8–110.9)
Reticulocyte count (%)	8.7 (3.7–15)
White blood cells count (10^3^/ml)	6.6 (4.6–8.8)
Absolute neutrophil count (10^3^/ml)	3572 (2318–5282)
Platelet count (10^3^/ml)	340 (204–448)

*ACS* acute chest syndrome; *IQR* Inter-quartile range; *SCD* sickle cell disease

^a^Others indicate poor growth, abnormal transcranial doppler, or cerebrovascular accident

### Perceptions of SCD/Hydroxyurea and participant characteristics

In our cohort, participants’ perceptions of SCD and hydroxyurea varied across B-IPQ domains by age, sex, and number of hospitalizations in the year prior to study enrollment (Table 2). Median scores indicated that older participants had more positive perceptions of personal control of SCD compared to younger ones (7 vs. 5, *p* = 0.04), while females perceived more SCD symptoms compared to their male counterparts (6 vs. 1, *p* = 0.02), respectively. None of the B-IPQ domain scores was significantly different by SCD genotype (data not shown).

**Table 2:** Perceptions of sickle cell disease and hydroxyurea in different patient groups (*N* = 34)

B-IPQ domains, median (IQR)	All (*N* = 34)	Age			Gender		
		12–17 years (*n* = 25)	18–22 years (*n* = 9)	*P*-value	Male (*n* = 20)	Female (*n* = 14)	*P*-value
Consequences	5 (1, 7)	5 (1, 7)	6 (3, 8)	0.50	3 (0, 7)	7 (4, 8)	0.15
Personal control	5 (3, 8)	5 (3, 8)	7 (6, 8)	**0.04**	5 (4, 9)	5 (3, 8)	0.61
Treatment control	8 (5, 10)	7 (6, 10)	9 (5, 10)	0.98	8.5 (6, 10)	7 (5, 9)	0.18
Identity (symptoms)	5 (0, 7)	5 (0, 7)	5 (1, 6)	0.81	1 (0, 6)	6 (4, 8)	**0.02**
Concerns	6 (2, 9)	5 (2, 9)	6 (5, 8)	0.59	5 (2, 9)	7 (4, 9)	0.55
Understanding	9 (8, 10)	8 (7, 10)	10 (9, 10)	0.09	9 (7, 10)	9.5 (8, 10)	0.52
Emotional response	3 (0, 8)	3 (0, 8)	7 (0, 10)	0.52	0.5 (0, 8)	7 (2, 8)	0.13

Data are presented as medians and inter-quartile ranges

*P*-value <0.05 was statistically significant (highlighted in bold)

*B-IPQ* brief illness perception questionnaire; *IQR* inter-quartile range

Higher B-IPQ scores indicated worse perceptions of sickle cell disease related consequences, identity or disease-related symptoms, concerns, and emotional response, but better perceptions of personal control, treatment control, and understanding of sickle cell disease

### Perceptions of SCD/Hydroxyurea and healthcare utilization

When compared to those with 1–3 or no hospitalizations, participants with 4 or more hospitalizations perceived more SCD-related consequences (8 vs. 5 vs. 1, *p* < 0.001) and symptoms (8 vs. 5 vs. 0, *p* < 0.001) as well as worse emotional response to SCD (10 vs. 7 vs. 0, *p* < 0.001), and they also perceived hydroxyurea as less beneficial in controlling their SCD (5 vs. 9 vs. 10, *p* = 0.01) (Table 3). Longer total length of stay (LOS) significantly correlated with more perceived SCD-related symptoms (*r*_s_ = 0.72, *p* < 0.001), consequences (*r*_s_ = 0.73, *p* < 0.001), concerns (*r*_s_ = 0.34, *p* = 0.04), and worse emotional response to SCD (*r*_s_ = 0.75, *p* < 0.001). Longer LOS also correlated significantly with less perceived personal control of SCD (*r*_s_ = −0.34, *p* = 0.05) and benefits of hydroxyurea controlling their SCD (*r*_s_ = −0.54, *p* < 0.01). More frequent emergency department (ED) visits were associated with more perceived SCD-related symptoms (*r*_s_ = 0.68, *p* < 0.001), consequences (*r*_s_ = 0.68, *p* < 0.001), worse emotional response (*r*_s_ = 0.65, *p* < 0.001) and less perceived benefits of hydroxyurea controlling their SCD (*r*_s_ = −0.46, *p* = <0.01).

**Table 3:** Perceptions of sickle cell disease and hydroxyurea in relation to healthcare utilization (*N* = 34)

B-IPQ domains	ED visits in 1 year		Hospitalizations in 1 year, median (IQR)			
	r_s_ correlations	*P*-value	None (*n* = 14)	1–3 (*n* = 11)	≥ 4 (*n* = 9)	*P*-value
Consequences	0.68	**<0.001**	1 (0, 4)	5 (2, 7)	8 (7, 9)	**<0.001**
Personal control	– 0.28	0.11	7 (5, 10)	4 (2, 10)	5 (3, 5)	0.20
Treatment control	– 0.46	**<0.01**	10 (7, 10)	9 (5, 10)	5 (3, 7)	**0.01**
Identity (symptoms)	0.68	**<0.001**	0 (0, 1)	5 (4, 6)	8 (6, 10)	**<0.001**
Concerns	0.3	0.08	3 (0, 8)	5 (4, 8)	8 (6, 9)	0.11
Understanding	0.005	0.98	10 (7, 10)	9 (7, 10)	10 (8, 10)	0.72
Emotional response	0.65	**<0.001**	0 (0, 1)	7 (0, 8)	10 (8, 10)	**<0.001**

Data are presented as spearman correlations for ED visits, and as medians and inter-quartile ranges for hospitalizations categories

*P*-value <0.05 was statistically significant (highlighted in bold)

*B-IPQ* brief illness perception questionnaire; *ED* emergency department; *IQR* inter-quartile range

Higher B-IPQ scores indicated worse perceptions of sickle cell disease related consequences, identity or disease-related symptoms, concerns, and emotional response, but better perceptions of personal control, treatment control, and understanding of sickle cell disease

### Perceptions of SCD/Hydroxyurea and self-reported adherence

In our cohort, 25 (74%) participants reported low adherence to hydroxyurea using **©**MMAS-8 scale. Participants with higher **©**MMAS-8 scores, indicating greater adherence to hydroxyurea, perceived more benefits of hydroxyurea (*r*_s_ = 0.44, *p* < 0.01) and had better emotional response to SCD (*r*_s_ = −0.44, *p* = 0.01) (Table 4). Participants with moderate or high adherence to hydroxyurea perceived more benefits of hydroxyurea (10 vs. 7, *p* = 0.04), less symptoms (0 vs. 6, *p* = 0.03) and better emotional response related to SCD (0 vs. 7, *p* < 0.01), when compared to those with low hydroxyurea adherence.

**Table 4:** Participants’ perceptions of sickle cell disease and hydroxyurea in relation to self-report adherence levels (*N* = 34)

B-IPQ domains	©MMAS-8		©MMAS-8, median (IQR)		
	r_s_ correlations	*P*-value	Low (*n* = 25)	Mod/High (*n* = 9)	*P*-value
Consequences	−0.26	0.13	6 (2, 8)	4 (0, 5)	0.08
Personal control	0.31	0.07	5 (3, 7)	5 (5, 9)	0.26
Treatment control	0.44	**<0.01**	7 (5, 9)	10 (8, 10)	**0.04**
Identity (symptoms)	−0.3	0.08	6 (3, 8)	0 (0, 4)	**0.03**
Concerns	−0.04	0.8	6 (2, 8)	5 (4, 10)	0.94
Understanding	0.2	0.25	9 (7, 10)	10 (8, 10)	0.38
Emotional response	−0.44	**0.01**	7 (0, 10)	0 (0, 1)	**<0.01**

Data are presented as spearman correlations for MMAS-8 adherence scores, and as medians and inter-quartile ranges for the low and moderate/high MMAS-8 adherence categories

*P*-value <0.05 was statistically significant (highlighted in bold)

*B-IPQ* brief illness perception questionnaire; *IQR* inter-quartile range; **©** *MMAS-8* **©**Modified Morisky Adherence Scale 8-items; *r*_*s*_ spearman correlations

Higher ©MMAS-8 scores indicate better or higher adherence level to hydroxyurea

Higher B-IPQ scores indicated worse perceptions of sickle cell disease related consequences, identity or disease-related symptoms, concerns, and emotional response, but better perceptions of personal control, treatment control, and understanding of sickle cell disease

### Perceptions of SCD/Hydroxyurea and Laboratory Markers of Adherence

In a sub-group of HbSS patients, we examined the relationship of MCV and HbF, as additional surrogates for adherence, to B-IPQ scores. Participants with high MCV values reported less negative perceptions of SCD-related symptoms (1 vs. 6, *p* = 0.01), better emotional response (0 vs. 7, *p* < 0.01), and more perceived benefits of hydroxyurea (9 vs. 6, *p* = 0.01), compared to those with low MCV values (Table 5). We found no significant differences in participants’ perceptions of SCD and hydroxyurea using their median HbF level (16%) as a cutoff. However, using a HbF% cutoff of 10%, participants with low HbF% reported worse emotional response related to SCD (10 vs. 0, *p* < 0.001) and less perceived benefits of hydroxyurea (5 vs. 9, *p* = 0.04), when compared to those with high HbF%, respectively.

**Table 5:** Participants’ perceptions of sickle cell disease and hydroxyurea in relation to adherence laboratory markers in homozygous sickle cell patients (*N* = 29)

B-IPQ domains, median (IQR)	HbF%			MCV		
	Low <16% (*n* = 15)	High ≥16% (*n* = 14)	*P*-value	Low <102 (*n* = 15)	High ≥102 (*n* = 14)	*P*-value
Consequences	6 (1, 8)	5 (0, 7)	0.69	7 (2, 9)	3 (0, 5)	**0.05**
Personal control	5 (3, 10)	5 (4, 8)	0.82	5 (3, 7)	5 (7, 9)	0.32
Treatment control	7 (5, 9)	9 (7, 10)	0.15	6 (5, 8)	9 (7, 10)	**0.01**
Identity (symptoms)	6 (1, 8)	4 (0, 6)	0.2	6 (5, 8)	1 (0, 4)	**0.01**
Concerns	7 (4, 9)	5 (2, 9)	0.48	6 (4, 9)	5 (2, 8)	0.44
Understanding	10 (6, 10)	9 (8, 10)	0.83	10 (8, 10)	10 (7, 10)	0.65
Emotional response	7 (0, 10)	2 (0, 6)	0.1	7 (3, 10)	0 (0, 3)	**<0.01**

Data are presented as medians and inter-quartile ranges

*P*-value <0.05 was statistically significant (highlighted in bold)

*B-IPQ* brief illness perception questionnaire; *HbF* fetal haemoglobin level; *IQR* inter-quartile range; *MCV* mean corpuscular volume

Higher B-IPQ scores indicated worse perceptions of sickle cell disease related consequences, identity or disease-related symptoms, concerns, and emotional response, but better perceptions of personal control, treatment control, and understanding of sickle cell disease

Higher HbF % or MCV values indicate higher hydroxyurea adherence rates

### Perceptions of SCD/Hydroxyurea and HRQOL

About half of participants’ B-IPQ scores significantly correlated with different HRQOL domain scores as shown in Table 6. Participants with more SCD related consequences, concerns, and emotional response reported worse fatigue (*r*_s_ = 0.68, *p* < 0.001; *r*_s_ = 0.44, *p* = 0.01; *r*_s_ = 0.74, *p* < 0.001), pain (*r*_s_ = 0.56, *p* < 0.01; *r*_s_ = 0.54, *p* < 0.01; *r*_s_ = 0.63, *p* < 0.001), anxiety (*r*_s_ = 0.55, *p* < 0.01; *r*_s_ = 0.58, *p* < 0.001; rs = 0.56, *p* < 0.01), and depression (rs = 0.64, *p* < 0.001; rs = 0.49, *p* < 0.01; rs = 0.7, *p* < 0.001), respectively. In addition, participants with less perceived benefits of hydroxyurea reported worse fatigue (*r*_s_ = −0.6, *p* < 0.001), pain (*r*_s_ = −0.39, *p* = 0.03), anxiety (*r*_s_ = −0.47, *p* < 0.01), and depression (*r*_s_ = −0.62, *p* < 0.001).

**Table 6:** Participants’ perceptions of sickle cell disease and hydroxyurea in relation to their health-related quality of life using PROMIS® measures

B-IPQ domains	Fatigue (*N* = 31)	Pain interference (*N* = 31)	PF-UE (*N* = 31)	PF-Mobility (*N* = 31)	Anxiety (*N* = 31)	Depression (*N* = 31)	Peer relationships (*N* = 23)	Social Isolation (*N* = 8)
Consequences	0.68 **(<0.001)**	0.56 **(<0.01)**	**−**0.32 (0.08)	**−**0.59 **(<0.001)**	0.55 **(<0.01)**	0.64 **(<0.001)**	**−**0.1 (0.64)	0.55 (0.15)
Personal control	**−**0.28 (0.13)	**−**0.51 **(<0.01)**	0.2 (0.28)	0.2 (0.27)	**−**0.27 (0.14)	**−**0.3 (0.1)	0.02 (0.94)	**−**0.61 (0.1)
Treatment control	**−**0.6 **(<0.001)**	−0.39 **(0.03)**	0.36 **(0.04)**	0.59 **(<0.001)**	**−**0.47 **(<0.01)**	**−**0.62 **(<0.001)**	0.17 (0.43)	**−**0.81 **(0.01)**
Identity (symptoms)	0.56 **(<0.01)**	0.48 **(<0.01)**	**−**0.39 **(0.03)**	**−**0.52 **(<0.01)**	0.31 (0.09)	0.34 (0.06)	**−**0.07 (0.76)	0.32 (0.44)
Concerns	0.44 **(0.01)**	0.54 **(<0.01)**	**−**0.35 (0.06)	**−**0.35 (0.05)	0.58 **(<0.001)**	0.49 **(<0.01)**	**−**0.21 (0.33)	0.82 **(0.01)**
Understanding	**−**0.23 (0.21)	**−**0.3 (0.11)	0.1 (0.59)	0.1 (0.6)	0.13 (0.5)	**−**0.004 (0.99)	0.18 (0.42)	**−**0.08 (0.85)
Emotional response	0.74 **(<0.001)**	0.63 **(<0.001)**	**−**0.37 **(0.04)**	**−**0.6 **(<0.001)**	0.56 **(<0.01)**	0.7 **(<0.001)**	**−**0.13 (0.55)	0.74 **(0.03)**

Data are presented as spearman correlations and *p*-values in parentheses (*p*-value)

*P*-value <0.05 was statistically significant (highlighted in bold)

*B-IPQ* brief illness perception questionnaire; *IQR* inter-quartile range; *PF* physical function; *PROMIS*® Patient Reported Outcomes Information System; *UE* Upper Extremity

Note: Adolescent patients (12–17 years old) completed peer relationships PROMIS® measure (*n* = 23), while young adults (≥ 18 years old) completed social isolation PROMIS® measure (*n* = 8)

Higher PROMIS® domain scores indicated worse severity for fatigue, pain interference, depression, anxiety, and social isolation, but better physical functioning (mobility or upper-extremity), and peer relationships

Higher B-IPQ scores indicated worse perceptions of sickle cell disease related consequences, identity or disease-related symptoms, concerns, and emotional response, but better perceptions of personal control, treatment control, and understanding of sickle cell disease

## Discussion

With increasing efforts to expand access to hydroxyurea in paediatric and adult patients with SCD [4], understanding patients’ perceptions of SCD and hydroxyurea has gained importance. Our study contributes to the emerging literature on hydroxyurea by being the first to examine the relationship of patients’ perceptions of SCD and hydroxyurea to domains of HRQOL and hydroxyurea adherence amongst AYA with SCD. In our cohort, participants’ perceptions of SCD and hydroxyurea varied by age, sex and number of hospitalizations. Importantly, participants with lower adherence to hydroxyurea, both by self-report and by laboratory evidence, perceived less benefits of hydroxyurea, reported more negative perceptions of their symptoms and had worse emotional response to SCD. We also found that participants with more negative perceptions of SCD-related consequences, concerns, and emotional response, as well as fewer perceived benefits of hydroxyurea reported worse fatigue, pain, anxiety, and depression domain scores on HRQOL testing.

In our cohort, we found that patient perceptions of more SCD-related symptoms and consequences, worse emotional response to SCD, and lower perceived benefit of hydroxyurea, were associated with more frequent hospitalizations and ED visits. Similarly, earlier studies showed that parental perceptions of SCD severity and illness-related stress were associated with increased healthcare utilization [41, 42]. Perceptions related to disease severity have also been found to differ between patients and their caregivers, including physicians. Connelly et al. showed this discrepancy in their study, in which patients with SCD reported fewer SCD-related symptoms and milder disease severity compared to that reported by their parents and physicians [43]. However, in our study, we did not examine SCD perceptions among different informants.

Medication non-adherence rates are estimated to be 50–75% among paediatric patients with chronic health conditions, including SCD [13, 16], with lower adherence among adolescents [44]. A recent meta-analysis reported lower adherence rates in older patients and those with more identified barriers [16], which are numerous and varied in the literature [13–22]. Nevertheless, the relationship between patients’ perceptions of both hydroxyurea and SCD and their adherence to hydroxyurea has not been previously examined. In our study, we supplemented our evaluation of patients’ adherence and perceptions using the **©**MMAS-8 and B-IPQ scales, respectively, with traditional laboratory markers of adherence. Majority of our patients reported low adherence to hydroxyurea using the **©**MMAS-8. Patients with lower hydroxyurea adherence by self report and/or MCV level, but not HbF, perceived more negative effects of SCD in different domains and considered hydroxyurea a less beneficial treatment for SCD. Poor medication adherence may not be limited to hydroxyurea in SCD patients. Patel et al. reported that in patients with SCD, adherence to one medication was significantly associated with adherence to other medications, suggesting that personal perceptions drive adherence-related behavior within individuals regardless of the number of medications prescribed [15]. Moreover, a number of studies using the B-IPQ in other settings – asthma, hypertension, organ transplantation, and diabetes mellitus – have shown a significant relationship between patients’ and/or parental perceptions of their illness and its severity and medication adherence, using both self-report and laboratory markers [45–48].

Early reports have shown a relationship between patient and parental acceptance of hydroxyurea and their perceptions of SCD severity [20, 24–26], which may indirectly influence hydroxyurea adherence. Parents who perceived their children as having a milder form of SCD were less willing to accept the risk of hydroxyurea side effects, particularly in relation to birth defects and cancer risk [20, 24–26]. In contrast, parents of patients with severe SCD sought more information about hydroxyurea and were more accepting of its use as a preventive strategy [24, 25]. Different factors have been proposed to contribute to the decision of starting hydroxyurea in patients with SCD, including patients’ and parental perceptions. In a single-institution study, the majority of parents and patients were in favor of using hydroxyurea, which was perceived as safe and effective with balanced risks and benefits, compared to chronic transfusions and stem cell transplantation [49]. In our study, greater hydroxyurea adherence by patient self-report and higher MCV and HbF levels was associated with perceptions of greater treatment benefit from hydroxyurea, although the direction of any cause and effect relationship is not clear.

We also sought to examine patients’ perceptions of SCD and hydroxyurea in relation to their domains of HRQOL. Similar to children with other chronic conditions, children with SCD have poor HRQOL domain scores [3, 34, 36, 50]. In our cohort, patients’ perceptions correlated with different HRQOL domain scores. Patients with more negative perceptions of SCD-related consequences, concerns, and emotional response, or with less perceived benefits of hydroxyurea, reported worse fatigue, pain, anxiety, and depression scores. These relationships suggest an association between worse perceptions of SCD and/or hydroxyurea, and poor HRQOL domain scores. Nevertheless, given the nature of our cross-sectional study, we cannot determine the direction of the relationship, namely whether poor HRQOL leads to worse perceptions or that worse perceptions leads to poor HRQOL domain scores. Consistent with our results, O’Donovan et al. have shown that, in a cohort of patients with congenital heart disease, illness perceptions were also predictive of different HRQOL domains and psychological outcomes, including depression and anxiety [51].

Some limitations of our study warrant discussion. First, our study was at a single institution and data were collected from a convenience sample of SCD patients, which could limit the generalizability of our results. However, our study helped to generate further hypotheses that could inform a larger prospective study. Second, the cross-sectional design of the study limited our ability to examine the changes in patients’ perceptions, hydroxyurea adherence, and different domains of HRQOL over time. Third, we limited our study population to AYA population with a relatively narrow age range (12–22 years old). However, we focused on AYA because they represent a specific age group when patients start to take responsibility of their illness and be in charge of taking their hydroxyurea on their own, which make them at higher risk for low hydroxyurea adherence. Fourth, we evaluated SCD-related events (e.g. ED visits and hospitalizations) at our institution only and it is possible that SCD-related events elsewhere could have been missed. Finally, we used B-IPQ and **©**MMAS-8 to evaluate patients’ perceptions of SCD and hydroxyurea adherence, respectively, and both have not been validated for adolescents or in SCD. To address this, we conducted pre-testing to ensure participants’ comprehension of different items of the B-IPQ and **©**MMAS-8. Both measures have been used in published studies that included patients with SCD and/or other chronic conditions [14, 27, 47, 52–54], and in particular, the B-IPQ has been used in studies with patients as young as 8 years of age [27]. In addition, objective laboratory data supported patients’ perceptions and adherence levels by self-report using B-IPQ and **©**MMAS-8.

## Conclusions

Perceptions of disease and treatment amongst AYA with SCD correlated with subjective and objective adherence measures, HRQOL domain scores and number of hospitalizations. Our findings enhance our understanding of patients’ perceptions of SCD and hydroxyurea and suggest that hydroxyurea adherence is a multifactorial process. Understanding patients’ perspectives could support efforts to overcome adherence and utilization barriers. [55, 56] Given the fact that hydroxyurea adherence is a dynamic and multi-factorial process, changes in patients’ perceptions of hydroxyurea and how it helps them maintain control of their illness could serve as surrogate markers for early changes in hydroxyurea adherence over time. Therefore, our results suggest that a longitudinal prospective assessment of patients’ perceptions may reveal modifiable factors associated with early changes in hydroxyurea adherence and HRQOL over time. Routine assessment of patients’ perceptions of hydroxyurea and SCD, adherence to hydroxyurea, and HRQOL in SCD outpatient settings should be considered using different platforms. Given the wide access to technology [21, 57, 58], web- or mobile-based platforms could be utilized to allow the completion of different assessments more frequently, and in-person or remotely in different settings.

## Abbreviations

**©**MMAS-8: **©**Modified Morisky Adherence Scale 8-items
AYA: Adolescents and young adults
B-IPQ: Brief Illness Perceptions Questionnaire
CAT: Computerized Adaptive Testing
HbF: Fetal hemoglobin
HRQOL: Health-related quality of life
MCV: Mean corpuscular volume
PROMIS®: Patient Reported Outcomes Measurement Information System
SCD: Sickle cell disease
